# Social Connection Planning for Lonely or Socially Isolated Older Adults

**DOI:** 10.1093/geroni/igab046.135

**Published:** 2021-12-17

**Authors:** Emily Bower, Aurora Newman, Paige Reohr, Kimberly Vandorden

**Affiliations:** 1 Pacific University, Hillsboro, Oregon, United States; 2 University of Rochester Medical Center, Rochester, New York, United States

## Abstract

Social connections are important for maintaining health and well-being with age. Behavioral interventions to promote connectedness hold promise, but there is limited evidence to guide effective modifications in the context of physical distancing or quarantine restrictions, such as those required during the COVID-19 pandemic. We present evidence for a brief (1-2 session) social connection intervention, “Connections Planning,” to enhance social connectedness for older adults. We first describe a cognitive-behavioral model of loneliness, which served as the framework for developing the intervention. We then present two case examples to demonstrate the application of the intervention with older adults in a community mental health clinic during physical distancing restrictions. Finally, we present initial findings from a pilot study to examine the feasibility and acceptability of the intervention delivered remotely with up to 10 community-dwelling older adults who endorse clinically significant loneliness. Recommendations for adapting the intervention during physical distancing restrictions are provided.

